# Effects of Nanosecond Pulsed Electric Field on Immune Checkpoint Receptors in Melanoma Cells

**DOI:** 10.3390/ph16101362

**Published:** 2023-09-27

**Authors:** Natalia Sauer, Wojciech Szlasa, Anna Szewczyk, Vitalij Novickij, Jolanta Saczko, Dagmara Baczyńska, Małgorzata Daczewska, Julita Kulbacka

**Affiliations:** 1 Faculty of Pharmacy, Wroclaw Medical University, 50-556 Wroclaw, Poland; natalia.sauer@outlook.com; 2 Faculty of Medicine, Wroclaw Medical University, 50-556 Wroclaw, Poland; wojciech.szlasa@outlook.com; 3 Department of Molecular and Cellular Biology, Faculty of Pharmacy, Wroclaw Medical University, 51-618 Wroclaw, Poland; a.szewczyk@umw.edu.pl (A.S.); dagmara.baczynska@umw.edu.pl (D.B.); 4 Department of Animal Developmental Biology, Faculty of Biological Sciences, University of Wroclaw, Sienkiewicza 21, 50-335 Wroclaw, Poland; malgorzata.daczewska@uwr.edu.pl; 5 Institute of High Magnetic Fields, Vilnius Gediminas Technical University, 08217 Vilnius, Lithuania; vitalij.novickij@vilniustech.lt; 6 Department of Immunology, State Research Institute Centre for Innovative Medicine, Santariškių 5, 08410 Vilnius, Lithuania

**Keywords:** pulsed electric field, melanoma, PD-1, LAG-3, TIM-3, checkpoint receptors

## Abstract

Checkpoint molecules such as PD-1, LAG-3, and TIM-3 are currently under extensive investigation for their roles in the attenuation of the immune response in cancer. Various methods have been applied to overcome the challenges in this field. This study investigated the effects of nanosecond pulsed electric field (nsPEF) treatment on the expression of immune checkpoint molecules in A375 and C32 melanoma cells. The researchers found that the nsPEF treatment was able to enhance membrane permeabilization and morphological changes in the cell membrane without being cytotoxic. We found that the effects of nsPEFs on melanoma included (1) the transport of vesicles from the inside to the outside of the cells, (2) cell contraction, and (3) the migration of lipids from inside the cells to their peripheries. The treatment increased the expression of PD-1 checkpoint receptors. Furthermore, we also observed potential co-localization or clustering of MHC class II and PD-1 molecules on the cell surface and the secretion of cytokines such as TNF-α and IL-6. These findings suggest that nsPEF treatment could be a viable approach to enhance the delivery of therapeutic agents to cancer cells and to modulate the tumor microenvironment to promote an antitumor immune response. Further studies are needed to explore the mechanisms underlying these effects and their impacts on the antitumor immune response, and to investigate the potential of nsPEF treatment in combination with immune checkpoint inhibitors to improve clinical outcomes for cancer patients.

## 1. Introduction

Melanoma is a skin cancer that derives from the pigment-producing cells melanocytes [1,2,3]. It is known to be an immunogenic cancer, meaning that the immune system recognizes and responds to melanoma cells [4,5]. However, melanoma cells can evade the immune system by upregulating immune checkpoint receptors, which can suppress the function of immune cells and lead to immune tolerance and tumor progression [6,7]. While early-stage melanoma is often treatable with surgery, advanced melanoma can be extremely difficult to treat and often leads to poor outcomes [8]. In recent years, the development of immune checkpoint inhibitors has provided a promising new avenue for the treatment of metastatic melanoma [9].

Immune checkpoint receptors are a group of molecules which are expressed on the surfaces of immune cells and cancer cells and that regulate immune responses [10,11]. These receptors play a crucial role in balancing the activation and suppression of the immune system. The immune checkpoint receptors include molecules such as programmed cell death protein 1 (PD-1), T-cell immunoglobulin and mucin domain-containing protein 3 (TIM-3), and lymphocyte activation gene 3 (LAG-3), among others [12,13,14]. These receptors bind to ligands expressed on other cells, leading to the modulation of immune cell activity [15]. Expression of these receptors on cancer cells can inhibit the activity of immune cells such as T cells, which generally recognize and kill cancer cells [16,17]. By interacting with these immune checkpoint receptors, cancer cells can reduce the immune system’s ability to recognize them as foreign and evade destruction.

Programmed cell death protein-1 (PD-1) is a protein receptor found on the surface of immune cells, particularly T cells. However, it was found that PD-1 may be expressed on melanoma cells and that it plays a crucial role in promoting immune suppression and immune evasion [14,18]. Its expression on melanoma cells is associated with a more aggressive tumor phenotype [18]. Cancer cells can exploit the PD-1 pathway to evade detection and destruction by the immune system. In clinical practice, the interaction between PD-1 and its ligand, programmed death-ligand 1 (PD-L1), has emerged as a promising approach for treating melanoma [19,20].

LAG-3, or lymphocyte activation gene 3, is a protein expressed on the surface of certain immune cells and cancer cells [11]. LAG-3 plays a negative regulatory role in tumor immunology by binding to its ligands, including the major class II tissue compatibility complex (MHC II). The LAG3–MHC II interaction can reduce T-cell proliferation and protect cancer cells from drug-induced apoptosis [21]. In melanoma, LAG-3 is often overexpressed, which can inhibit the immune response via the suppression of T-cell activation and cytokine secretion, making the tumor more difficult for the organism to repress [22].

TIM-3 is primarily known as a protein expressed on the surface of immune cells, where it plays a role in regulating the immune response [23,24,25]. Recent studies have shown that TIM-3 can be expressed on the surface of melanoma cells, and its expression has been associated with poorer prognosis and resistance to certain immunotherapies [13]. The expression profile of TIM-3 on melanoma cells is variable and can depend on various factors, including the stage and progression of the disease, as well as the tumor microenvironment [13,26]. Studies have shown that TIM-3 expression is generally low on normal melanocytes but, in advanced or metastatic disease, can be highly upregulated on melanoma cells [13].

Modulating the expression of immune checkpoint receptors such as PD-1, TIM-3, and LAG-3 on melanoma cells can affect cancer by altering the interactions between the cancer cells and the immune system [27,28]. Upregulation of these immune checkpoint receptors on melanoma cells can suppress the function of T cells and natural killer cells, leading to immune tolerance and tumor progression [29,30,31,32]. Conversely, the downregulation of these immune checkpoint receptors on melanoma cells can enhance the function of T cells and natural killer cells, leading to an enhanced antitumor immune response. As immune checkpoint receptors such as LAG-3, PD-1, and TIM-3 offer a promising new approach for the treatment of advanced melanoma, we focused on finding a new tool to modulate their expressions. Electric fields have been utilized in various forms of cancer treatment [32,33,34,35,36,37,38]. Certain methods use radiofrequency or microwave technologies to raise the temperature of the tumor above 43 degrees Celsius, killing the cells through hyperthermia [39,40]. Meanwhile, other methods utilize pulsed electric fields (PEFs) to create pores in the tumor cells, which allows for the introduction of harmful drugs or DNA [41]. Ultrashort electrical pulses offer an alternative form of electrical cancer treatment that can modify the expression of surface antigens in cancer cells, making them more vulnerable to targeted therapies and increasing their recognition by the immune system [42,43]. In our study, our primary objective was to investigate the effects of nanosecond pulsed electric fields (nsPEFs) specifically on melanoma cells. We aimed to assess whether nsPEFs have the potential to selectively modulate the expression of immune checkpoint receptors in melanoma cells and to explore the involvement of major histocompatibility complex (MHC) molecules, which are crucial to antigen presentation to T cells within the context of melanoma. By examining these aspects, we aimed to gain insights into the potential of nsPEFs as a therapeutic intervention for melanoma, specifically targeting immune checkpoint regulation and the MHC-mediated immune response. The research sought answers to the future application of PEFs as an adjunct to cancer-targeted therapies or modulation of the immune system against melanoma.

## 2. Results

### 2.1. Biophysical Characterization of nsPEF-Treated C32 and A375 Cells

**Figure 1:**
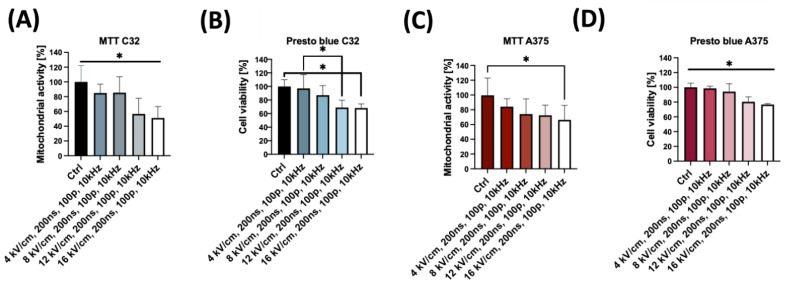
The effect of nsPEFs on melanoma cells’ viability and membrane permeabilization. Each experiment was performed independently three times, and we used triplicates for each experiment: (**A**) Viability of C32 cells after nsPEF treatment, determined by MTT assay after 24 h. The effect of nsPEF treatment was measured for four different electric field strengths. Graphs are representative of three independent experiments. Data are mean ± SD (n = 3 independent experiments). (**B**) PrestoBlue^TM^ assay was performed after the exposure of C32 cells to four different nsPEF strengths for 24 h. Graphs are representative of three independent experiments. Data are mean ± SD (n = 3 independent experiments). (**C**) Viability of A375 cells after nsPEF treatment, determined by MTT assay. The effect of the nsPEF was measured for four different electric field strengths. Graphs are representative of three independent experiments. Data are mean ± SD (n = 3 independent experiments). (**D**) PrestoBlue^TM^ assay was performed after the exposure of the A375 cells to four different strengths of nsPEF. Graphs are representative of three independent experiments. Data are mean ± SD (n = 3 independent experiments). * *p* < 0.05 in ANOVA test.

**Figure 2:**
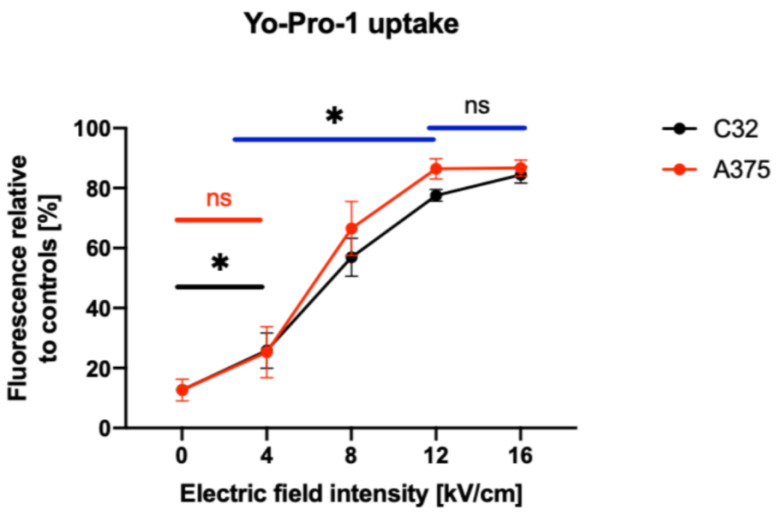
Permeabilization of C32 and A375 cells to YO-PRO-1 after being subjected to PEFs of various strengths. We increased the voltage parameters and observed the effects on membrane permeabilization. * *p* < 0.05 in ANOVA test, ns indicate no statistically significant differences.

We evaluated cell viability using MTT (Figure 1A,C) and PrestoBlue assays (Figure 1B,D), and measured the permeability of the cell membranes to YO-PRO-1 dye (Figure 2). The MTT and PrestoBlue assays revealed that the lowest electric field strength that significantly decreased the mitochondrial activity of the C32 cell line was 12 kV/cm with 200 ns pulse duration, 100 pulses, and a frequency of 10 kHz. In the case of the A375 cell line, a significant decrease was observed at 16 kV/cm with 200 ns pulse duration, 100 pulses, and a frequency of 10 kHz in the MTT assay, and at 12 kV/cm with 200 ns pulse duration, 100 pulses, and a frequency of 10 kHz in the PrestoBlue assay.

The most significant decrease in cell viability of the C32 cell line was observed between the control group and the group to which a 16 kV/cm electric field was applied. According to the results obtained from the MTT assay, the viability of the cells decreased from 100% in the control group to 51.11%. In the case of the PrestoBlue assay, the viability decreased from 100% in the control group to 68.28%. Similarly, in the A375 cell line, the most notable decrease in viability was also observed at a 16 kV/cm electric field strength for both the MTT assay, which showed a decrease from 100% to 66.33%, and the PrestoBlue assay, which showed a decrease from 100% to 76.75%.

We investigated the impact of nsPEF protocols on the permeabilization of melanoma cells and determined that there was no significant difference between the control group and the group to which a 4 kV/cm electric field was applied for the A375 cell line. However, statistically significant differences were observed when comparing the application of 4 kV/cm, 8 kV/cm, and 12 kV/cm electric field strengths. In contrast, for the C32 cell line, there was a significant difference between the control group and the group to which a 4 kV/cm electric field was applied, as well as between the 4 kV/cm, 8 kV/cm, and 12 kV/cm electric field groups. Interestingly, in both cell lines, there was no statistically significant difference between the 12 kV/cm and 16 kV/cm electric field groups.

**Figure 3:**
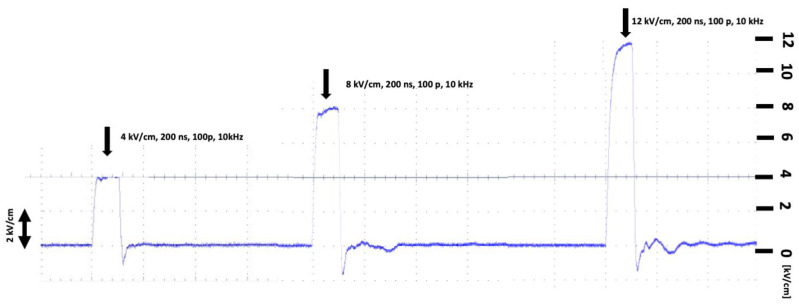
Shapes and parameters of electric pulses, captured using an oscilloscope.

Figure 3 shows the shapes and parameters of the electric pulses, captured using an oscilloscope.

### 2.2. The Effect of nsPEF Treatment on the Secretion of Cytokines

**Figure 4:**
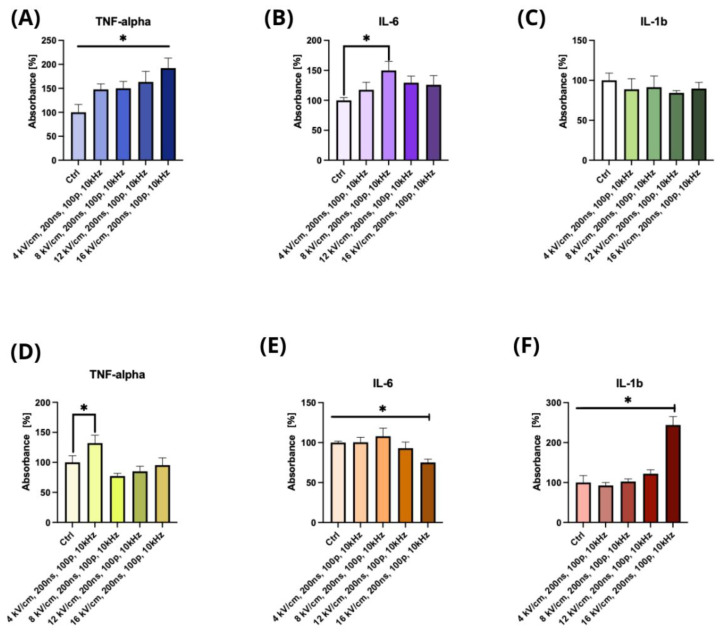
Immunoenzymatic assay of cytokine secretion in human melanoma cells post nsPEF exposure: (**A**) TNF-α secretion ELISA assay analysis after nsPEF treatment of A375 cells; (**B**) IL-6 ELISA assay analysis after nsPEF treatment of A375 cells; (**C**) IL-1b ELISA assay analysis after nsPEF treatment of A375 cells; (**D**) TNF-α secretion ELISA assay analysis after nsPEF treatment of C32 cells; (**E**) IL-6 ELISA assay analysis after nsPEF treatment of C32 cells; (**F**) IL-1b ELISA assay analysis after nsPEF treatment of C32 cells; * p < 0.05 in ANOVA test. Each experiment was performed independently three times, and we used triplicates for each experiment.

The ELISA results showed the impact of changing the parameters of the nsPEF treatment, including the application of 0, 4, 8, 12, and 16 kV/cm, with a duration of 200 ns, 100 p, and 10 kHz, on the A375 and C32 cell lines after 24 h of treatment (Figure 4). Figure 4A displays the secretion of TNF-α, which showed a statistically significant increase in absorbance between 0 and the other field strengths. Figure 4B displays the secretion of IL-6, which showed a significant increase in absorbance between 0 and 8 kV/cm. However, no statistically significant changes were observed for IL-1b, as shown in Figure 4C. For the C32 cell line, Figure 4D displays the secretion of TNF-α, which showed a statistically significant increase in absorbance between 0 and 4 kV/cm, whereas Figure 4E shows the secretion of IL-6, for which a significant decrease was observed between 16 kV/cm and the other field strengths. Finally, Figure 4F displays the secretion of IL-1b, which showed a statistically significant increase in absorbance observed between each lower field strength and 16 kV/cm. These results indicate that nsPEF treatment has a differential impact on cytokine secretion in the A375 and C32 cell lines, with some field strengths resulting in significant changes.

### 2.3. The Effect of nsPEF Treatment on the Expression of Immune Checkpoint Receptors and the Secretion of Cytokines

**Figure 5:**
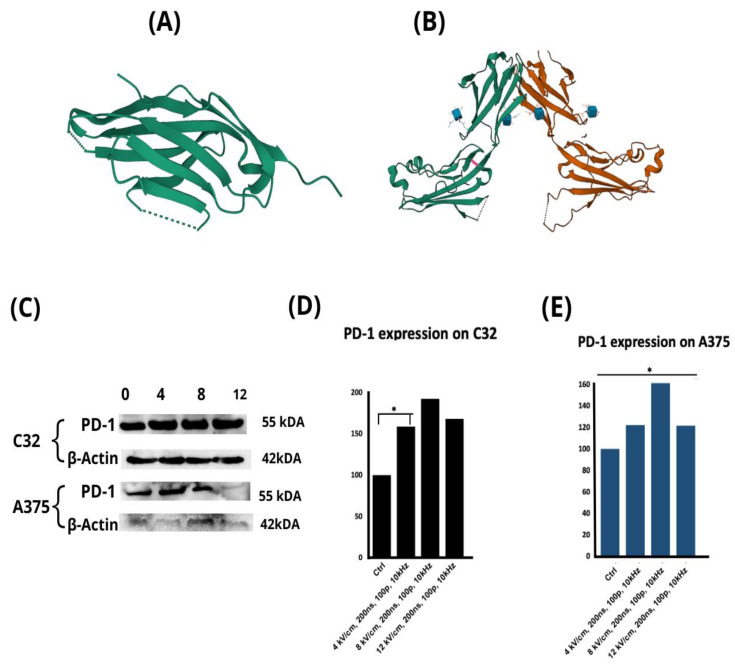
Molecular structures of (**A**) PD-1 antigen (PDB: 3RRQ) [44] and (**B**) TIM-3 antigen (PDB: 2OYP) [45]. Western blot analysis of PD-1 antigen expression in C32 and A375 cells following 0–12 kV/cm, 200 ns, 100p, 10 kHz nsPEF exposure after 24 hours of treatment (**C**), and densitometric analysis of the WB results in (**D**) C32 cells and (**E**) A375 cells. Statistical analysis of Western blot data: * p < 0.05 in ANOVA test.

PD-1 and TIM-3 are immune checkpoint receptors commonly co-expressed in melanoma cells. The molecular structures of these proteins are visualized in Figure 5A,B, respectively. Before this study, we hypothesized that exposure to an nsPEF might induce alterations in their expressions. Figure 5C shows the Western blot analysis of the expressions of these antigens. The statistical analysis revealed an elevation of the PD-1 protein expression in both C32 and A375 cell lines. In the case of the C32 cell line, statistically significant differences were found between the data collected for the 0 kV/cm and 4 kV/cm conditions (*p* = 0.025) (Figure 5D). In turn, in the case of the A375 cell line, statistically significant differences were observed between the 4 kV/cm and 8 kV/cm conditions (*p* = 0.017), the 4 kV/cm and 12 kV/cm conditions (*p* = 0.007), and the 0 kV/cm and 8 kV/cm conditions (*p* = 0.009) (Figure 5E). However, the expression levels of LAG-3 and TIM-3 proteins were rather low, and, although a visible increase was observed in the expression levels of these antigens in the C32 cell line, it was insufficient to obtain a statistically reliable evaluation. These data are included in the Supplementary Materials (Figure S1).

### 2.4. Morphological Changes of Cell Membranes following Treatment with nsPEFs

**Figure 6:**
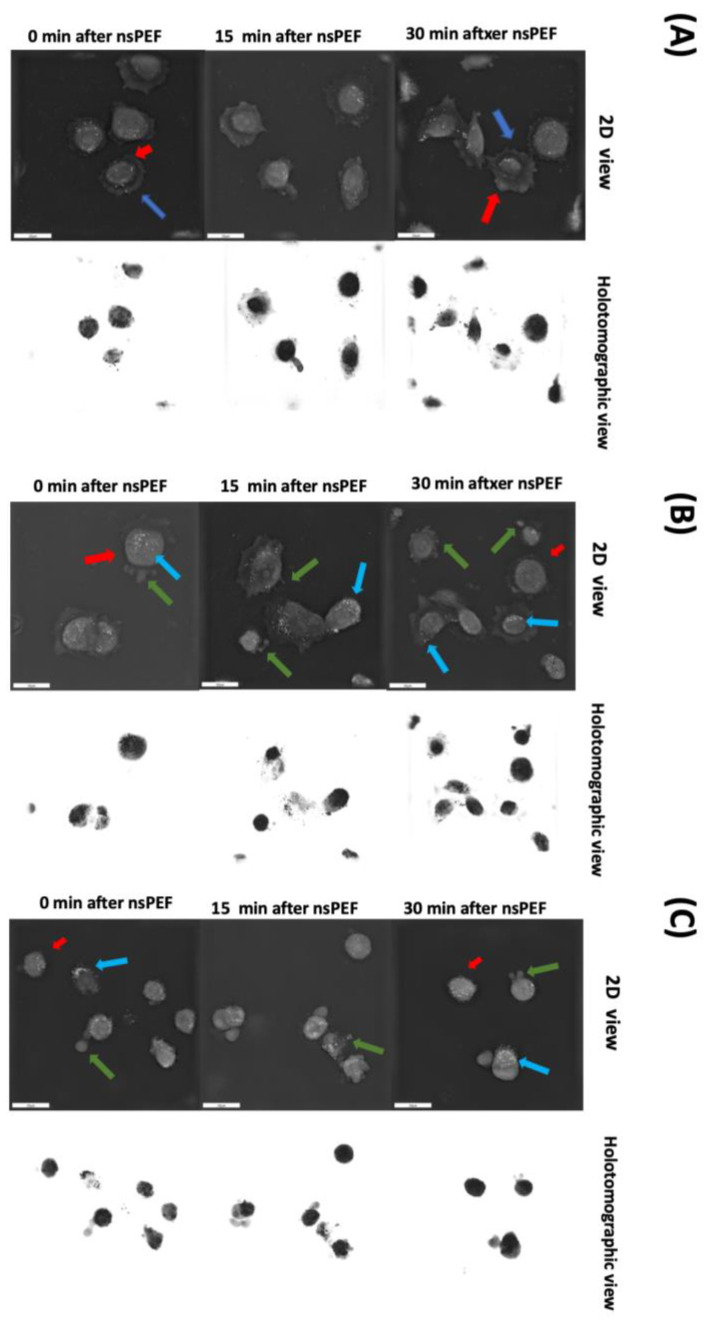
Holotomographic microscopy imaging of C32 cells. (**A**) Control cells after 0, 15, and 30 min; (**B**) cells after 4 kV/cm, 200 ns, 100 pulse, 10 kHz PEF treatment; (**C**) cells after 8 kV/cm, 200 ns, 100 pulse, 10 kHz PEF treatment. Red arrows indicate cellular contraction, blue arrows show the shift in the distribution of lipids within the cells, and green arrows indicate the blebbing and vesiculation observed in the nsPEF-treated cells.

To assess the effects of nsPEF treatment on cellular morphology, we utilized holotomography with a 4 kV/cm, 200 ns, 100 pulse, 10 kHz PEF treatment (Figure 6B) and an 8 kV/cm, 200 ns, 100 pulse, 10 kHz PEF treatment (Figure 6C), and compared these treatment groups with control cells (Figure 6A). We observed that control cells did not exhibit any changes in their size, while nsPEF-treated cells experienced contraction (indicated by red arrows). This effect was observed 30 min after applying an nsPEF at 4 kV/cm, and immediately after applying an nsPEF at 8 kV/cm. Additionally, we noticed that lipids were uniformly distributed within healthy cells and were closer to the centroid. However, after administering nsPEF at both strengths, lipids (indicated by white droplets) began to shift toward the cell periphery. The morphology of the control cells remained unchanged, whereas the nsPEF-treated cells exhibited a more elliptical shape, which was evidenced in the 4 kV/cm treatment condition after 30 min and in the 8kV/cm treatment condition immediately after nsPEF application. We also observed that nsPEF-treated cells began to undergo blebbing and vesiculation (indicated by green arrows).

**Figure 7:**
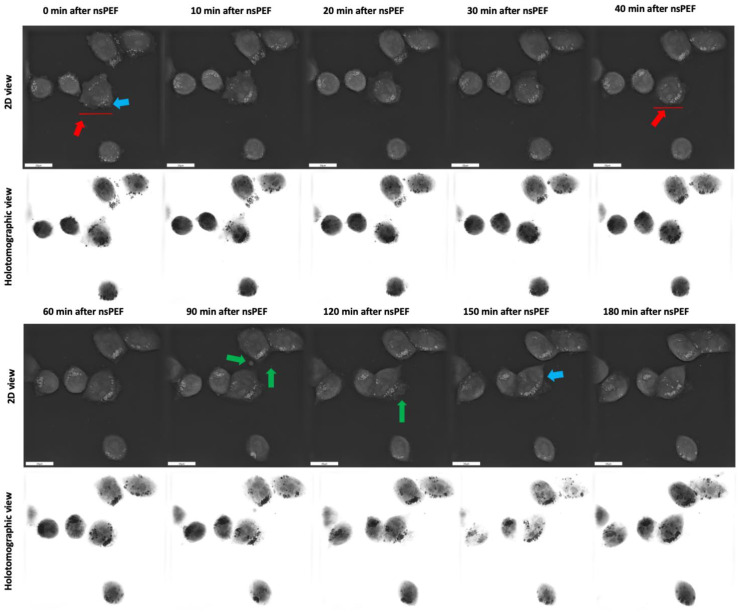
Holotomographic microscopy imaging of C32 cells after 8 kV/cm, 200 ns, 100 pulse, 10 Hz PEF treatment. Red arrows indicate cellular contraction, blue arrows indicate the shift in the distribution of lipids within the cells, and green arrows indicate the blebbing and vesiculation observed in the nsPEF-treated cells.

In order to further investigate the effects of the 8 kV/cm, 200 ns, 100 pulse, 10 kHz nsPEF treatment on C32 cell morphology, we observed the cells for 180 min (Figure 7). Firstly, we observed the extracellular release of cytoplasmic contents and damage to the plasma membrane, as shown by the green arrows. However, the extent of blebbing was not as pronounced as that observed for the 12 kV/cm nsPEF treatment. Secondly, we observed an increase in the number of lipid-containing droplets rearranging towards the cell periphery, as indicated by blue arrows. Finally, we observed cell contraction, as indicated by red arrows. Additionally, the cells underwent changes in shape from a typical irregular shape with pseudopodia to a more elliptical shape. All of the described changes intensified over time.

**Figure 8:**
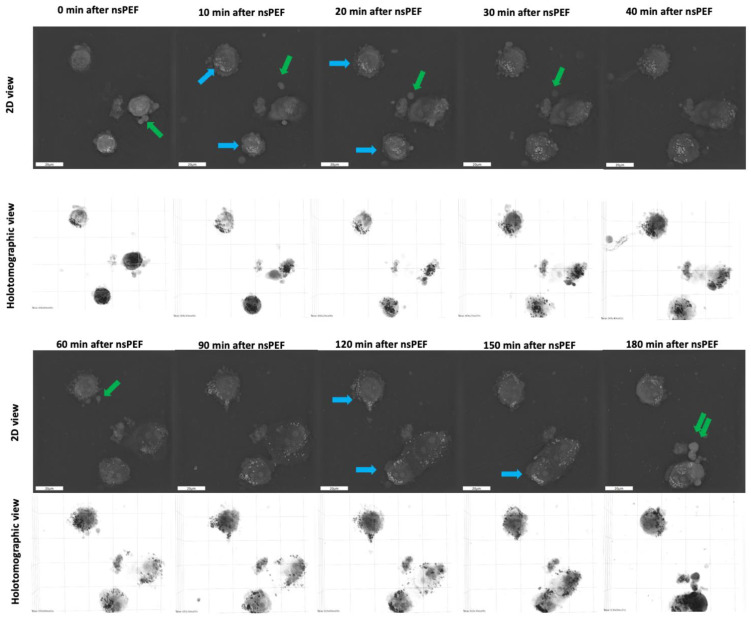
Holotomographic microscopy imaging of A375 cells after 8 kV/cm, 200 ns, 100 pulse, 10 kHz PEF treatment, blue arrows indicate the shift in the distribution of lipids within the cells, and green arrows indicate the blebbing and vesiculation observed in the nsPEF-treated cells.

Next, we applied the same nsPEF parameters to the A375 cell line (Figure 8). Cells subjected to the 8 kV/cm, 200 ns, 100 pulse, 10 kHz treatment also exhibited blebbing and released vesicles outside the cells (green arrows). The vesicle secretion was more pronounced than in the C32 cell line. The blue arrows indicate that the lipids in this nsPEF-treated cell line also began to arrange peripherally and migrate from the cell interiors to the edges.

These data suggest that the effect of nsPEF on melanoma cell lines leads to reproducible changes: (1) induction of vesicles from the inside to the outside of the cells; (2) cell contraction; and (3) the migration of lipids from inside the cells to their peripheries. These changes intensified over time.

### 2.5. MHC Class II and PD-1 Expression

**Figure 9:**
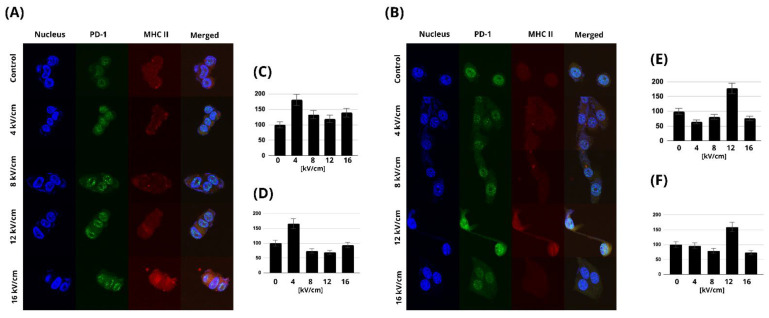
Confocal microscopy studies were used to investigate how the expression profiles of PD-1 and MHC-II changed after nsPEF treatment: (**A**) Fluorescent signals of PD-1 and MHC-II after 24 h nsPEF treatment of C32 cells; (**B**) fluorescent signals of PD-1 and MHC-II 24 h after the nsPEF treatment of A375 cells. In addition to utilizing DAPI to observe the nuclei (blue), we employed Alexa Fluor 488 to mark PD-1 (green) and Alexa Fluor 546 to identify MHC class II (red) in both A375 and C32 cells. (**C**) The fluorescent signal intensity of PD-1 in the C32 cell line; (**D**) the fluorescent signal intensity of MHC II in the C32 cell line; (**E**) the fluorescent signal intensity of PD-1 in the A375 cell line; (**F**) the fluorescent signal intensity of MHC II in the A375 cell line.

We performed confocal microscopy studies to investigate how the expression profiles of PD-1 and MHC-II changed after nsPEF treatment. Across the analyzed data, the most substantial rise in expression was observed for the 4 kV/cm, 200 ns, 100 p, 10 kHz nsPEF treatment of C32 cells (Figure 9A). In comparison, for cells of the A375 line, the highest expression levels were observed after the 12 kV/cm, 200 ns, 100 p, 10 kHz nsPEF treatment (Figure 9B). Additionally, we observed the formation of aggregations and red and green dots in the fluorescence images. The aggregations appeared as clusters of fluorescent signals, while the red and green dots were small, distinct puncta of fluorescence. The aggregations and puncta were observed in images labeled with AlexaFluor 546, which targets MHC class II, and in images labeled with AlexaFluor 488, which targets PD-1. This suggests a potential co-localization or clustering of MHC class II and PD-1 molecules on the cell surface following nsPEF treatment.

## 3. Discussion

Cancer immunotherapy has revolutionized cancer treatment in the past decade, and immune checkpoint inhibitors have shown great promise in treating various cancers [46]. However, the efficacy of immune checkpoint inhibitors is not universal across all patients, and some patients may not respond at all [46]. Therefore, there is a need to explore alternative approaches to enhance the efficacy of immune checkpoint inhibitors. One such approach is the use of nanosecond pulsed electric fields (nsPEFs), which have been shown to enhance the permeability of the cell membrane and improve the delivery of therapeutic agents [47,48,49,50]. In this study, we investigated the effects of nsPEF treatment on C32 and A375 cells’ biophysical characteristics, morphology, and expression of immune checkpoint receptors.

Our findings indicate that the 4–8 kV/cm, 200 ns, 100 pulse, 10 kHz protocol has a high potential to enhance membrane permeabilization while showing minimal cytotoxicity to the cells, as demonstrated using the MTT and PrestoBlue assays. This is a different effect compared to microsecond electrical impulses, where the cytotoxic effect can reach up to 50% (when using 2 kV/cm pulses, and up to ~10% when using 4 kV/cm pulses) [48].

Additionally, we observed morphological changes in the cell membranes, such as the extracellular release of cytoplasmic contents and plasma membrane damage, increased lipid-containing droplets, and cells undergoing shrinkage. Interestingly, a study by Szlasa et al. confirmed that tumor cells treated with nsPEFs exhibit similar morphological changes [49]. Additionally, the latest study demonstrated that ultrashort electrical pulses could be used to induce tumor suppression.

Moreover, we observed an increase in the expression levels of PD-1 and MHC-II and potential co-localization or clustering of MHC class II and PD-1 molecules on the cell surface following nsPEF treatment. The ELISA results demonstrated that nsPEF treatment had a significant impact on the secretion of cytokines. Specifically, there was a significant increase in the secretion of TNF-α and IL-6, whereas no significant changes were observed for IL-1b. These findings suggest that nsPEF treatment could modulate the tumor microenvironment by inducing the secretion of pro-inflammatory cytokines, which could promote an antitumor immune response. Similar effects were also observed in vivo, where nsPEF showed inhibitory effects on malignant melanoma proliferation [49]. In addition to an increase in TNF-α, an increase in IL-2 and a decrease in IL-10 were also observed.

The use of nsPEFs to enhance the delivery of therapeutic agents has been widely investigated in recent years. Morphological changes in the cell membrane may affect the interactions between immune checkpoint receptors and their ligands. For instance, the increase in the expression of PD-1 and MHC-II following nsPEF treatment could enhance the antitumor immune response by increasing T-cell activation. This observation is consistent with the results of previous studies showing that the upregulation of PD-1 and MHC-II is associated with improved clinical outcomes in patients treated with immune checkpoint inhibitors [50]. A study by Johnos et al. showed that MHC-II expression contributes to sensitivity to PD-1/PD-L1 axis inhibition [51]. It was observed that there was slower tumor formation and prolonged survival in the subgroup of melanoma cells with the highest degree of MHC-II positivity that were treated with anti-PD-L1. It is known that 60–67% of patients do not respond to anti-PD-1 pharmacotherapy [28,47,52].

Integrating nsPEF treatment into therapy may offer a potential solution to the issue at hand. By upregulating PD-1 expression in melanoma cells, we can create more targets for targeted therapy, and by increasing MHC II expression, we can intensify the immune response, thereby allowing more drugs to reach the tumor site selectively. Although the occurrence of PD-1 on T cells as an immune checkpoint molecule has been proven to reduce T-cell activity during immune responses to prevent autoimmune tissue damage, little is known about the role of this molecule when it occurs on cancer cells. A study by Kleffel et al. revealed that PD-1 inhibition on melanoma cells typically suppresses tumor growth in immunocompetent, immunocompromised, and PD-1-deficient tumor transplant recipient mice [14]. At the same time, many studies report that it is possible to inhibit the growth of melanomas using nanosecond electric pulses [43,53,54]. Since we know from in vivo studies that nanosecond electrical pulses do not indue aggressiveness and can even inhibit melanoma, it is worthwhile to further explore this tool as an inducer for target delivery to targeted therapies that may selectively attack melanoma cells. 

Our research also discovered an elevated level of TNF-α following nsPEF treatment, which is of particular interest since Lim et al. demonstrated that this cytokine upregulates PD-L1 in cancer cells [53,53]. [Interestingly, PD-1-positive patients frequently co-express the PD-1 molecule. It is noteworthy that combining LAG-3 and PD-1 blockade exhibits better clinical efficacy than monoclonal anti-PD-1 therapy [55]. Dual-targeted therapy may offer numerous benefits, including the upregulation of effector T-cell activity, leading to the inhibition of tumor growth [56]. Moreover, it may serve as a solution for the treatment of cases resistant to single-antibody therapy [57]. However, our results indicate that LAG-3 and TIM-3 expression levels are low in melanoma cells, making it harder to observe changes in expression. Thus, further research in this area is crucial to determine whether these immune checkpoint molecules’ expression levels play a meaningful role in the context of melanoma.

Another important aspect of our work is the possibility of modulating the secretion of pro-inflammatory cytokines through the stimulation of melanoma cells by nsPEF. Enhanced levels of IL-6, TNF-α, and IL-1b in melanoma can trigger an immune response. Some studies have shown that the activated PD-1/PD-L1 axis correlates with the upregulated presence of those cytokines [58,59]. However, this correlation has not been well studied in melanoma cell lines, and further research is needed to understand the relationship better.

The role of inflammation in cancer remains a subject of debate due to the dual nature of its effects, with both tumor-promoting and tumor-suppressive properties being reported. In the context of melanoma, IL-1B has been demonstrated to enhance the antitumor capabilities of T helper 1 (Th1) cells [60]. Moreover, it has been shown that IL-1 acts synergistically with IFN-γ to induce antitumorigenic activity in tumor-infiltrating macrophages. The increased IL-6 levels observed in metastatic melanoma patients may have a beneficial role. Studies have indicated that in patients undergoing ICI (immune checkpoint inhibitor) therapy for malignant melanoma, a lower baseline level of IL-6 is strongly linked to the development of immune-related adverse events such as colitis [61,62]. In vivo, the advantageous impacts of high TNF-α levels were predominantly observed when the cytokine was locally administered at relatively high concentrations and in repeated doses. This cytokine demonstrated its ability to hinder tumor growth by inflicting damage on the tumor vasculature and directly inducing cancer cell apoptosis. These effects were particularly pronounced in cases where NF-κB and JNK activation were impaired [63,64,65,66]. Furthermore, TNF-α exhibited the potential to enhance the effectiveness of cancer treatment drugs/chemotherapy by promoting increased blood vessel permeability [67].

Through in vitro experiments, we have demonstrated promising results that can contribute to the development and creation of therapeutic interventions. Additionally, our research enhances the understanding of the underlying mechanisms by which nsPEFs influence checkpoint molecule expression. Based on our results, we can suppose that the use of nsPEFs might be useful for (1) modulation of cell morphological features, which may contribute to the inhibition of tumor progression; (2) upregulation of PD-1 expression, which is a target for precision therapies; and (3) possible boosting of the immune response through the modulation of secreted cytokines. However, further investigation and validation of the efficacy and safety of these nsPEF protocols are required. Our results can support the planning of future in vivo studies, which will contribute to a more comprehensive understanding of the protocols’ effectiveness and facilitate the translation of these findings into clinical practice.

## 4. Materials and Methods

### 4.1. Cell Culture

The investigation was carried out utilizing two cell lines, namely C32 and A375. C32, an amelanotic melanoma cell line (ATCC^®^ CRL-1585™, LGC Standards, Kielpin, Poland), was derived from the skin of a 53-year-old male Caucasian patient, while A375, a melanotic melanoma cell line (ATCC^®^ CRL-1619™, LGC Standards, Poland), was derived from the skin of a 54-year-old female patient. The cells were grown as monolayers in 75 mL polystyrene cell culture flasks (Falcon^®^, Corning Life Sciences, Tewksbury, MA, USA) and cultured in Dulbecco’s modified Eagle’s medium (DMEM, Sigma-Aldrich, St. Louis, MO, USA) supplemented with 10% fetal bovine serum (FBS, Sigma-Aldrich) and antibiotics (streptomycin/penicillin). The cells were incubated at 37 °C in a humidified atmosphere with 5% CO_2_ (Hereus^®^, Thermo Fisher, Scientific, Waltham, MA, USA), and were washed with PBS and removed using trypsinization (0.025% trypsin and 0.02% EDTA; Sigma-Aldrich) as needed. The cells used in our experiments were regularly tested for *Mycoplasma* contamination, and no contamination was detected during the course of the study. Regarding the passages of the cells used in our experiments, the cells were used up to the seventh passage.

### 4.2. MTT Viability Assay

The cells’ viability was evaluated by assessing their mitochondrial activity through an assay. Cells were trypsinized and exposed to pulsed electric field protocols. Cells were then seeded onto 96-well plates (SARSTEDT AG & Co. KG, Nümbrecht, Germany) at a density of 3 × 10^4^ cells. After 24 h, cell viability was assessed based on the standard protocol provided by the manufacturer. The culture medium was removed from each well, and a solution of 0.5 mg/mL MTT (3-(4,5-dimethylthiazol-2-yl)-2,5-diphenyltetrazolium bromide, Sigma-Aldrich) in PBS buffer was added to make up a volume of 100 μL. The samples were then incubated at 37 °C for 2 h, following which acidified isopropanol (100 μL, 0.04 M HCl in 99.9% isopropanol) was added to dissolve the formazan crystals. The samples were mixed thoroughly by pipetting to ensure complete dissolution. The absorbance of each well was measured at 570 nm using a multiplate reader (GloMax, Promega, Walldorf, Germany). The results were expressed as the percentage of viable cells relative to untreated control cells.

### 4.3. PrestoBlue™ Viability Assay

Cell viability was assessed using PrestoBlue™ reagent (A13261, Invitrogen™, Carlsbad, CA, USA) 24 h after exposure to nsPEFs. The cells were cultured on 96-well plates at a density of 3 × 10^4^ cells and incubated for 30 min with the PrestoBlue™ reagent. The fluorescence measurements were taken at 520 nm excitation and 580–600 nm emission wavelengths using a multiplate reader (GloMax, Promega, Madison, WI, USA). All experiments were repeated in triplicate, and measurements included a blank sample.

### 4.4. Electric Field Treatment

Cells were suspended in electroporation cuvettes with an electrode gap of 1 mm (BTX, Syngen Biotech, Wrocław, Poland) during the pulse delivery procedure. The cells were detached from culture flasks via trypsinization (0.025% trypsin and 0.02% EDTA; Sigma-Aldrich) and then suspended in a cuvette with an SHM buffer (10 mM HEPES (Sigma-Aldrich), 250 mM sucrose (Chempur), and 1 mM MgCl_2_ (Sigma-Aldrich) in milliQ water) at a count of 10,000 cells per well. Then, the samples were exposed to PEFs. For the electroporation, a square wave electroporator (100 ns to 1 ms) was used to administer electric pulses (Institute of High Magnetic Fields, VGTU, Vilnius, Lithuania). Upon completion of the procedure, the electroporation buffer was replaced with a culture medium (DMEM) and the cells were seeded onto culture plates.

### 4.5. YO-PRO-1 Uptake Studies

The permeabilization of the melanoma cells in response to PEF treatments was analyzed using flow cytometry (Cube-6, Sysmex EUROPE GmbH, Warsaw, Poland). The cells were detached from the culture flasks with trypsin, centrifuged, and suspended in electroporation SHM buffer (10 mM HEPES (Sigma-Aldrich), 250 mM sucrose (Chempur), and 1 mM MgCl_2_ (Sigma-Aldrich) in milliQ water). Cells were maintained in suspension and pulsed in a cuvette (VWR) with two aluminum plate electrodes (1 mm gap). Subsequently, the cells were treated with nsPEF protocols. Then, the cells were incubated for 1 min. In the next step, cells (10^6^/mL) were resuspended in 0.3 mL of PBS. Flow cytometry analysis was performed using a Cube-6 flow cytometer (Sysmex, Warsaw, Poland). The fluorescence of YO-PRO-1 was excited with a 488 nm laser and assessed using a FL-3 detector (700/50). The YO-PRO^®^-1 dye, a specialized green fluorescent carbocyanine nucleic acid stain, serves a crucial function in the identification of apoptotic cells. It selectively permeates apoptotic cells, distinguishing them from live cells which remain unstained. Furthermore, YO-PRO^®^-1 does not penetrate dead cells, making it a valuable tool alongside propidium iodide (P-3566), a stain for nonviable cells. Data were collected and analyzed using CyView software (Sysmex, Warsaw, Poland).

### 4.6. Holotomographic Microscopy Studies

Live holographic tomography was performed using a 3D Cell Explorer microscope (Nanolive SA, Ecublens, Switzerland). C32 and A375 cells were cultured on 35 mm Ibidi µ-Dish glass dishes (Ibidi GmbH, München, Bayern, Germany). Cells were then imaged for 60 min. During this short imaging period, sufficient CO_2_ was maintained using a CO_2_-independent culture medium (Sigma-Aldrich). The temperature was set at 37 °C and was controlled using an Ibidi heating system (Ibidi GmbH, München, Bayern, Germany). Photographs of the samples were taken and analyzed using Steve software (https://www.ncbi.nlm.nih.gov/pmc/articles/PMC8879905/ (accessed on 24 July 2023)) (Nanolive, Sygnis, Poland).

### 4.7. Confocal Microscopy

CLSM (confocal laser scanning microscopy) was used to visualize the MHC class II, LAG-3, and PD-1 expression levels and cell nuclei. Following the experiment, the cells were seeded onto microscope plates. After 24 h incubation, the cells were washed three times with PBS. Fluoroshield™ with DAPI (4,6-diamidino-2-phenylindole, Sigma-Aldrich) was used to visualize nuclei and to mount the cells. In the experiment, we stained the cells with anti-MHC Class II (CoraLite^®^647 MHC Class II (I-A/I-E) Monoclonal Mouse Antibody, CL647-65122, Proteintech, San Diego, CA, USA) at a dilution of 1:400 and anti-PD-1 (PD-1/CD279 Monoclonal Mouse Antibody, 66220-1-Ig, Proteintech) at a dilution of 1:400. As secondary antibodies, anti-mouse antibodies were used, conjugated respectively with AlexaFluor488™ (Thermo Fisher Scientific, Waltham, MA, USA) and AlexaFluor546™ (Thermo Fisher Scientific, Waltham, MA, USA) diluted (1:250) in 4% Triton X-100 in PBS. An Olympus Fluoview FV1000 confocal laser scanning microscope was used to capture the images. We optimized the cut-off values based on the control unstained samples. The images were analyzed using Fiji software (Version: 2.1.0/1.53C) to evaluate the expression patterns of the proteins in the cross-sections of the cells.

### 4.8. Western Blot

The study utilized the Western blot technique (WB) to measure the expression levels of PD-1, LAG-3, TIM-3, and β-actin in the A375 and C32 cell cultures. Protein lysates were obtained from the cell cultures using T-PER Tissue Protein Extraction Reagent (Thermo Fisher Scientific, Inc., Waltham, MA, USA) with the addition of Halt™ Protease Inhibitor Cocktail (Thermo Fisher Scientific, Inc.) and 0.2 mM PMSF (Sigma-Aldrich; Merck KGaA), and protein concentrations were quantified using the BCA Protein Assay (Pierce, Rockford, IL, USA). Equal amounts of total protein (100 µg) were mixed with Laemmli sample buffer (Bio-Rad, Warsaw, Poland) and subjected to SDS-PAGE on 10% acrylamide gels. The separated proteins were transferred to nitrocellulose membranes (Sigma-Aldrich, Poznan, Poland) using the Transblot Bio-Rad System, followed by blocking in a 4% bovine serum albumin solution in TBST buffer (0.2 M Tris; 1.5 M NaCl; 0.1% Tween-20). The membranes were then incubated overnight at 4 °C with primary rabbit and mouse anti-human antibodies—Anti-LAG-3 antibody (16616-1-AP, Proteintech), Anti-PD-1 (66220-1-Ig, Proteintech, USA), and Anti-TIM-3 (60355-1-Ig, Proteintech, San Diego, CA, USA)—and subsequently incubated with secondary HRP-conjugated donkey anti-rabbit and anti-mouse antibodies. The membranes were then treated with the Luminata Classico chemiluminescent substrate, and the resulting reactions were visualized using the ChemiDoc Imaging System (Bio-Rad, Warsaw, Poland). An internal control, β-actin, was detected using a primary rabbit anti-human β-actin antibody and a secondary HRP-conjugated donkey anti-rabbit antibody, and was used to normalize the number of antigens. A densitometric analysis of the results was performed using Image Lab software, and the blotting results were analyzed using ImageJ software (Version: 2.1.0/1.53C).

### 4.9. ELISA Assay

The secretory properties of the cells following nsPEF treatment were analyzed using a colorimetric protocol. After the experiment, the cells were transferred to plates (Nunc, Roskilde, Denmark) at a density of 20,000 cells per well and allowed to adhere for 24 h in a culture medium (DMEM, Sigma-Aldrich, St. Louis, MO, USA). The culture medium was collected from each well and stored at −20 °C. The medium was then transferred to a 96-well plate coated with antibodies specific for mouse IL-6 (clone MQ2-39C3, 13-7068-81) at a dilution of 1 µg/mL, TNF-α (clone Mab11, 14-7349-81) at a dilution of 1 µg/mL, and IL-1β (clone CRM56, 12-7018-41) at a dilution of 1 µg/mL. The wells were washed and a biotinylated anti-IL-6 antibody, anti-TNF-α monoclonal antibody, and anti-IL-1β monoclonal antibody were added, respectively. After washing away the unbound antibodies, HRP-conjugated streptavidin was pipetted into the wells. At the end, the stop solution (CNB0011, Invitrogen™, USA) was added to each well, causing the color to change from blue to yellow in proportion to the total amount of IL-6, TNF-α, or IL-1β in each well. The absorbance was measured at 450 nm and the data were plotted.

### 4.10. Statistical Analysis

The experiments were performed in three replicates. The results are reported as mean ± standard deviation. The significance of the differences between the mean values of different groups of cells were assessed via one-way and two-way ANOVA with the Tukey post hoc test. Differences between treated samples and control cells that reached *p* ≤ 0.05 were assumed to be statistically significant. The results were analyzed using Microsoft Office Excel 2017 and GraphPad Prism 8.0 software.

## 5. Conclusions

In conclusion, our study suggests that nsPEF treatment has the potential to enhance the efficacy of immune checkpoint inhibitors by modulating the tumor microenvironment. The observed increases in the expression of immune checkpoint receptors and the secretion of pro-inflammatory cytokines could promote an antitumor immune response, potentially improving clinical outcomes for cancer patients. These findings warrant further investigation into the use of nsPEFs in combination with immune checkpoint inhibitors in preclinical and clinical studies. Additionally, future studies could explore the mechanisms underlying the observed morphological changes in the cell membrane and their effects on immune checkpoint receptor–ligand interactions. Overall, the use of nsPEFs as a strategy to enhance cancer treatment represents an exciting avenue for future research.

## Data Availability

Data is contained within the article and Supplementary Material.

## Supplementary Materials

The following supporting information can be downloaded at https://www.mdpi.com/article/10.3390/ph16101362/s1, Figure S1: (A) Western blot analysis of LAG-3 antigen expression following 0-12kV/cm, 200ns, 100p, 10kHz nsPEF exposure in C32 and A375 cells, and (B) molecular structure of LAG-3 antigen (PDB: 7TZE) [39].
