# Supplementary material for: Effects of Nanosecond Pulsed Electric Field on Immune Checkpoint Receptors in Melanoma Cells
Source: Pharmaceuticals (Basel). 2023 Sep 27;16(10):1362. doi: 10.3390/ph16101362 (PMC10610193; doi:10.3390/ph16101362)
Supplement: Supplementary file 1 [file pharmaceuticals-16-01362-s001.zip › pharmaceuticals-2551138-supplementary.pdf]

Supplementary Materials

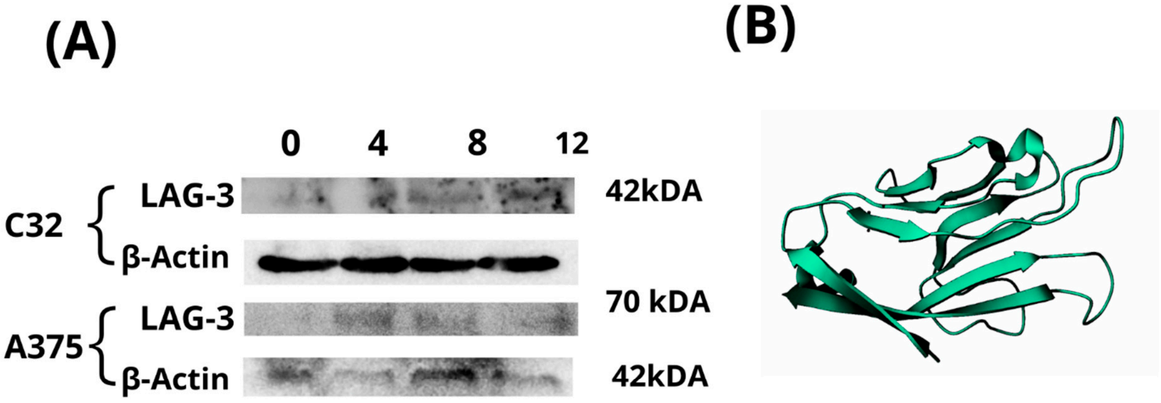

Figure S1. (**A**) Western Blot analysis of the LAG-3 antigen expression following 0-12kV/cm, 200ns, 100p, 10kHz nsPEF exposure on C32 and A375 cells, and (**B**) molecular structure of LAG-3 antigen (PDB: 7TZE) [39].
